# Neuron secrete exosomes containing miR-9-5p to promote polarization of M1 microglia in depression

**DOI:** 10.1186/s12951-022-01332-w

**Published:** 2022-03-09

**Authors:** Xian Xian, Li-Li Cai, Yang Li, Ran-Chao Wang, Yu-Hao Xu, Ya-Jie Chen, Yu-Hang Xie, Xiao-Lan Zhu, Yue-Feng Li

**Affiliations:** 1 Department of Radiology, Affiliated Hospital of Jiangsu University, No. 438, Jiefang Road, Zhenjiang, 212001 Jiangsu China; 2 Department of Central Laboratory, The Fourth Affiliated Hospital of Jiangsu University, No. 20, Zhengdong Road, Zhenjiang, 212001 Jiangsu China

**Keywords:** Neuroinflammation, Depression, Exosomes, miR-9-5p, Microglial polarization

## Abstract

**Background:**

Neuroinflammation is an important component mechanism in the development of depression. Exosomal transfer of MDD-associated microRNAs (miRNAs) from neurons to microglia might exacerbate neuronal cell inflammatory injury.

**Results:**

By sequence identification, we found significantly higher miR-9-5p expression levels in serum exosomes from MDD patients than healthy control (HC) subjects. Then, in cultured cell model, we observed that BV2 microglial cells internalized PC12 neuron cell-derived exosomes while successfully transferring miR-9-5p. MiR-9-5p promoted M1 polarization in microglia and led to over releasing of proinflammatory cytokines, such as interleukin-1β (IL-1β), interleukin-6 (IL-6) and tumor necrosis factor-alpha (TNF-α), which exacerbated neurological damage. Furthermore, we identified suppressor of cytokine signaling 2 (SOCS2) as a direct target of miR-9-5p. Overexpression of miR-9-5p suppressed SOCS2 expression and reactivated SOCS2-repressed Janus kinase (JAK)/signal transducer and activator of transcription 3 (STAT3) pathways. Consistently, we confirmed that adeno-associated virus (AAV)-mediated overexpression of miR-9-5p polarized microglia toward the M1 phenotype and exacerbated depressive symptoms in chronic unpredictable mild stress (CUMS) mouse mode.

**Conclusion:**

MiR-9-5p was transferred from neurons to microglia in an exosomal way, leading to M1 polarization of microglia and further neuronal injury. The expression and secretion of miR-9-5p might be novel therapeutic targets for MDD.

**Graphical Abstract:**

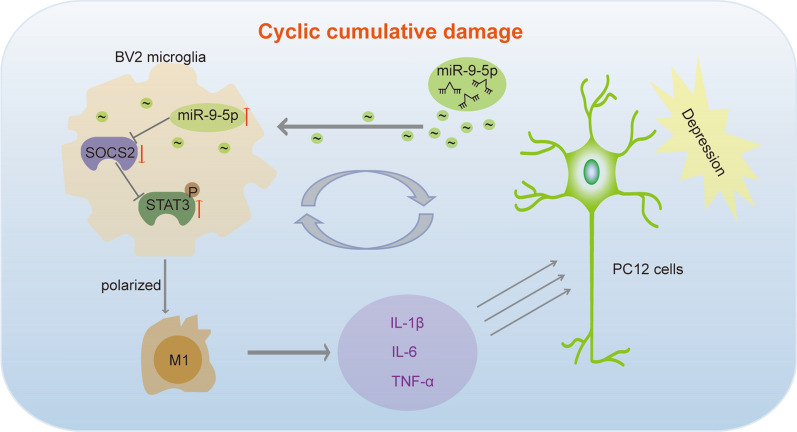

**Supplementary Information:**

The online version contains supplementary material available at 10.1186/s12951-022-01332-w.

## Background

An estimated 350 million people worldwide are impacted by depression (also known as major depressive disorder, MDD), a multifaceted mood disorder which associated to both environmental and genetic factors [1]. Depression can lead to adverse outcomes, such as incapacity, poor health behaviors, increased mortality and morbidity and increased use of health services [2]. Approximately 40% of patients failed to effectively respond to currently available treatments, which might ought to lack of mechanistic understanding of MDD development and the occurrence of suicidal behavior [3]. The correlation between psychiatric disorders and neuroinflammation was reported decades ago: increased levels of inflammatory markers often occurred in patients with depression, while the presence of depressive symptoms was positively associated with patients suffering from chronic inflammatory disorders [4, 5]. A series of longitudinal experiments in animals and humans have elaborated that neuroinflammation and inflammation, which are relevant biological factors interacting with neurophysiological mechanisms and external stimuli, are tightly associated to depression deteriorate [6]. On the other hand, the “inflammation hypothesis” of MDD was also supported by clinical findings that anti-inflammatory drugs have therapeutic efficacy in reducing depressive symptoms [7]. Microglia participated in the initiation and progression of the brain-derived inflammatory microenvironment [8]. It was reported that immune cell microglia were highly activated in the brains of patients with depression, indicating that neuroinflammation played a key role in pathogenic mechanisms of the depressed brain [5]. Recently, more studies made rapid progress in understanding the neurobiology of astrocytes and microglia, but the role of microglia-neuron interactions in MDD is unclear.

Microglia are classified into two subtypes: classically activated (M1) cells, which secrete proinflammatory cytokines, and alternatively activated (M2) cells, which promote tissue regeneration and repair [9–12]. M1 (iNOS-positive) macrophages were critical inflammatory contributors to neurodegenerative patients with MDD [13]. In physiological status, microglia were commonly in the resting M2 (CD206-positive) state in the central nervous system (CNS) [14]. Secretion of IL-1β, IL-6 and TNF-α with macrophage-associated proinflammatory cytokines was synchronized with M1 activation in MDD patients [15]. In addition, microglia not only assisted cytokines production in immune response but also played a vital role in controlling synaptic interactions, neurogenesis and neuronal cell death [5].

Growing evidence showed that secreted extracellular vehicles (EVs), including exosomes and ectosomes ranging in size from 50 to 200 nm, were important carriers in mediating intercellular signaling. Their cargos varied from miRNAs, mRNAs to proteins [16]. Transmission of exosomes to central neurons was mediated by microglia, astrocytes and oligodendrocytes, which could help propagating disease rather than positively supporting neurons [17]. Interestingly, recent studies reported that depression patients generated miRNAs targeting immune cells and altering neurotrophic factors expression, in addition to some key pathways to synaptic plasticity, memory and learning [5]. A typical example was that exosomal miR-139-5p acted as a negative regulator in neuronal differentiation and neural stem cell (NSC) proliferation, and its downregulation alleviated depressive-like behavior in CUMS mice [18].

In this study, we discussed the role of neuroinflammation in MDD pathogenesis and progression. The process of neuroinflammation dynamically switched microglia states in stress response, fulfilling its etiological role in depressive-like behavior. We explored the biological function and underlying mechanism of key miRNAs transferred by neuronal-derived exosomes in microglial polarization in MDD. We also proposed a potential exosome-based therapeutic strategy for neurodegenerative and mental disorders.

## Results

### MDD patient serum- derived exosomes promoted microglia M1 polarization

**Fig. 1:**
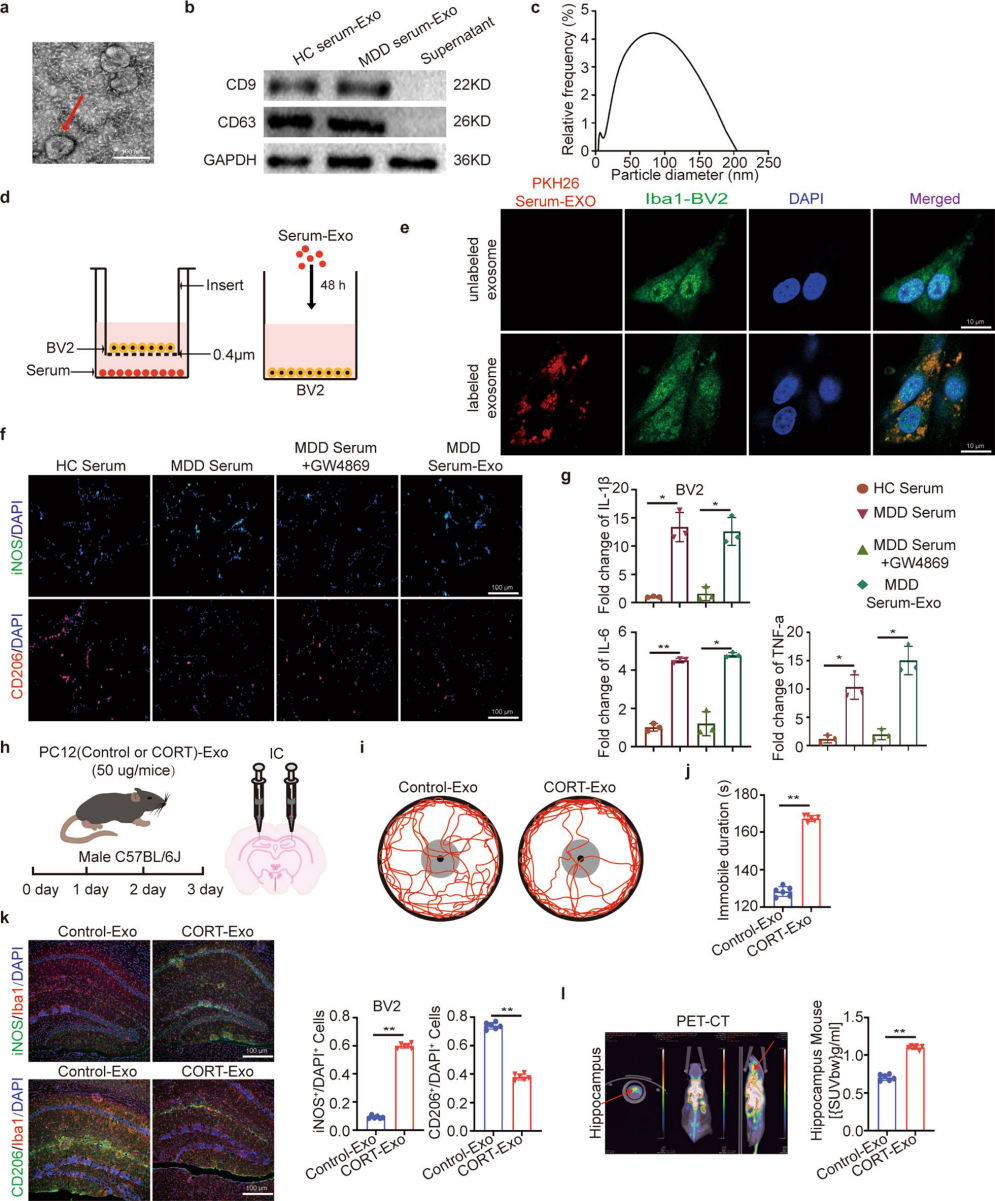
Serum-derived exosomes from depression patients induced microglia M1 polarization. Serum exosomes isolation, identification and uptake. **a**–**c** Exosomal characterization was profiled with electron microscopy, western blotting and NTA. **d** Schematic diagram of serum or serum exosomes co-culture with BV2 cells. **e** Internalization of PKH-26-labeled exosomes was analyzed in BV2 cells cocultured with serum exosomes. Scale bar = 10 µm. **f** iNOS^+^ and CD206^+^ staining for microglia in indicated four groups. Scale bar = 100 µm. n = 3. **g** Related fold change of IL-1β, IL-6 and TNF-α mRNA in microglia treated as described above. Their relative expression levels were measured by 2^−ΔΔCt^. **h** Flow chart of the animal experiment. **i** Representative tracks depicting mice in the Control-Exo and Cort-Exo groups during OFT. The red lines represent animal move path. The black dot represents the center of the arena. The black circle represents the outer boundary of the arena. The gray circle represents the boundary of the center arena area (see “Materials and methods”section). n = 6. **j** Immobile duration of mice in the two groups was recorded. **k** (Left) after exosomes injection, C57BL/6 J mice were sacrificed for immunofluorescence staining in the hippocampus of brain tissue. Scale bar = 100 µm. (Right) the proportions of M1 and M2 polarized microglia are shown in the statistical plots. **l** (Left) the representative PET/CT images. (Right) accumulation of [^18^F]DPA-714 in the hippocampus

Based on the fact that the activation and polarization of microglia are regulated by the brain-derived microenvironment [19], we first isolated exosomes from MDD patients, and examined their function on microglia polarization. Images acquired by transmission electron microscope (TEM) showed isolated exosomes as circular double-layered vesicles, ranging from 50 to 200 nm in diameter (Fig. 1a). We observed high expression of the exosomal markers CD9 and CD63 in the isolated samples, but undetectable in the remaining serum supernatant (Fig. 1b). By Nano Particle Tracking and Zeta Potential Distribution Analyzer (NTA), we further measured the accurate size of vesicles, which were approximate 80 nm in diameter (Fig. 1c). Based on these evidences, we validated the identity of isolated exosome samples.

The ability of lipopolysaccharide (LPS) to induce microglial activation has been broadly validated [20, 21]. As expected, Iba1-labeled microglia turned into round shape in morphology after LPS stimulation (Additional file 1: Fig. S1a). To verify whether the microenvironment was another causal factor of microglial polarization, serum and exosomes isolated from MDD patients were cocultured with BV2 cells (Fig. 1d). PKH-26-labeled exosomes were incorporated to the cytoplasm of recipient cells (Fig. 1e). Then, we measured the ratio of microglia in M1/ M2 phase, by labeling them with iNOS and CD206 respectively [22–24]. Compared to control group, the proportion of iNOS^+^ cells in total cells were significantly increased in the groups cocultured with MDD serum or exosomes (Fig. 1f, Additional file 1: Fig. S1b). Increased activation of microglia was reported as a main inducer for inflammatory factors releasing [13]. As shown in Fig. 1g, we observed M1 microglia exhibited high expression of inflammatory factors, including IL-1β, IL-6, and TNF-α, whereas effectively inhibited the polarization of BV2 cells upon exosome biogenesis inhibitor GW4869 treatment. In summary, MDD patients-derived serum and serum exosomes had similar function on promoting M1 polarization in microglia, but the effect of serum exosomes was more pronounced.

### Exosomes derived from MDD cell model induced microglial polarization in vivo and in vitro

Next, we extended the research of MDD patient-derived exosomes in vivo. We adopted a widely used MDD cellular model with corticosterone (CORT) [25], and tested it by measuring cell viability upon a series of different concentration of CORT treatments. We found 200 μM CORT reduced approximately half survival rate comparing to control group, and chose this dose for subsequent experiments (Additional file 1: Fig. S1c).

To confirm whether exosomes derived from cultured PC12 cells could be traced by fluorescence imaging, and whether the fluorescence intensity measurement could be used for estimating exosome amount, we labeled exosomes with the fluorescence marker DiR in vitro. Results indicated a strong linear correlation between exosomal concentration and signal intensity (Additional file 1: Fig. S1d). Similarly, in the in vivo validation experiment, fluorescence signals were captured every other day after intravenous injection of exosomes by near-infrared fluorescence imaging (NIRFI). We noted that on days 1 and 3, the mean radiation efficiency was significantly higher in the DiR-labeled exosome group than unlabeled group (Additional file 1: Fig. S1e). Furthermore, NIRF images clearly displayed exosomal signals in the liver, which disappeared by day 14 (Additional file 1: Fig. S1f), indicating that exogenously-imported exosomes had been completely metabolized. Since neural exosomes have been shown able to across the blood–brain barrier (BBB) [26–30], we collected brain tissue sections from mice for examination. We saw exosomes derived from PC12 cells were also transported across the BBB into the brain by being able to detect PKH26-labeled exosomes (Additional file 1: Fig. S1g). To assess the effect of cultured cell-derived exosomes on behavior change, exosomes were stereotaxically injected into the mouse hippocampus (Fig. 1h). Comparing to control group, exosomes treated mice showed depressive-like behavior, as evidenced by significant avoidance of the central part of the arena in the open field test (OFT) and increased immobility in the forced swimming test (FST) (Fig. 1i, j, Additional file 1: Fig. S1h). The proportion of iNOS^+^ cells was also increased in the treated group (Fig. 1k). [^18^F]DPA-714 signal was reported to indicate microglial activation and neuroinflammation in the hippocampus [31]. We also observed increased mean [^18^F]DPA-714 uptake in the hippocampus (Fig. 1l), indicating an increased expression of neuroinflammatory signals in our experimental setting [32]. These results confirmed that exosomes from a MDD cellular model promoted M1 polarization and exacerbated depressive-like behaviors and neuroinflammation.

### miR-9-5p was identified as a potential functional exosomal cargo in miRNA microarray

**Fig. 2:**
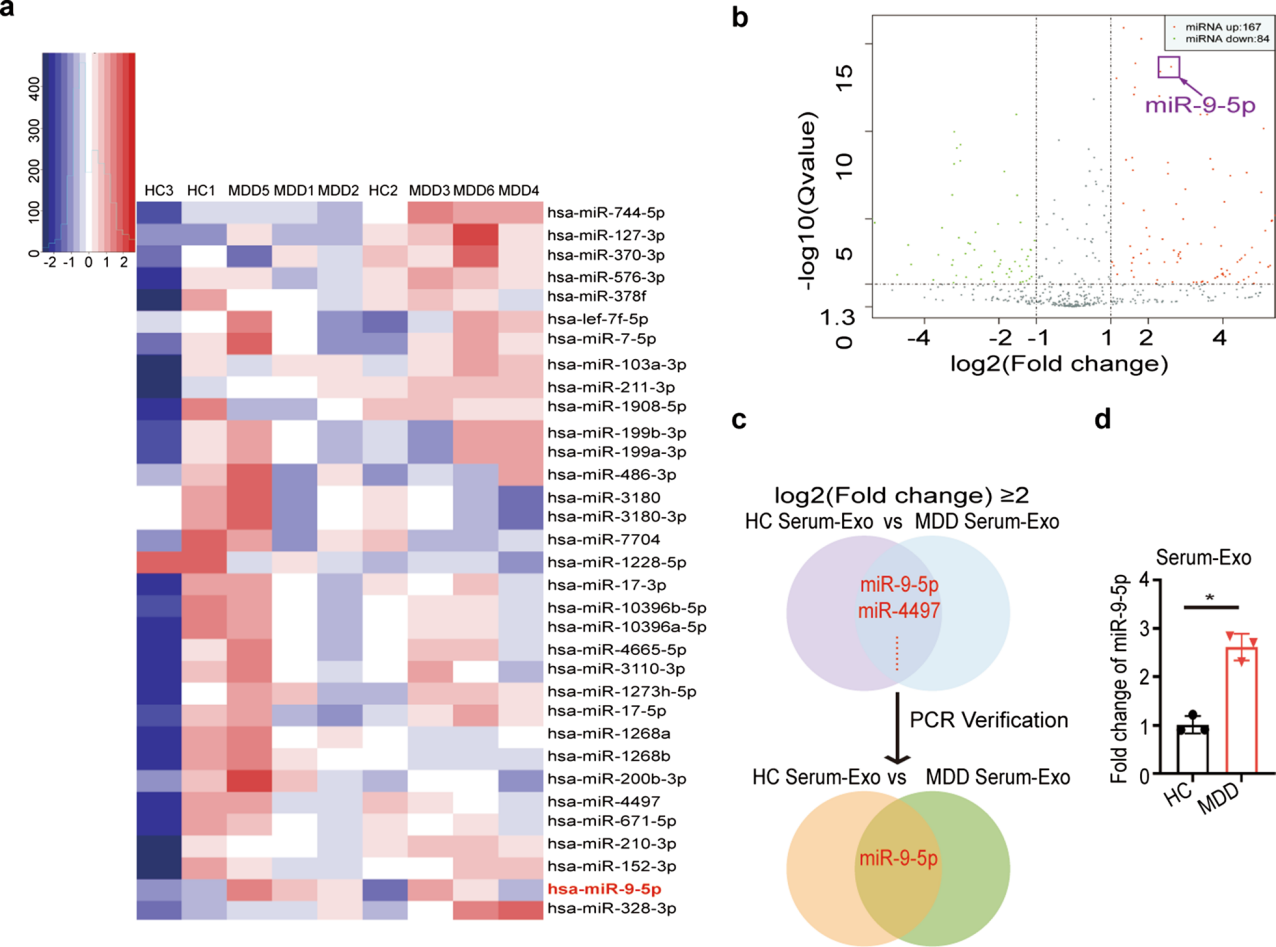
miRNA microarray analysis and qRT-PCR validation for serum exosomes derived from depression patients.** a** Partial heatmap of differentially expressed serum exosomal miRNAs between MDD patients and HC subjects. **b** Volcano maps showed that there were 167 miRNAs with higher expression and 84 miRNAs with lower expression in the serum exosomes of MDD patients compared to HC subjects. **c** Venn diagram showed that miR-9-5p was a miRNA with high expression in the serum exosomes of MDD patients. **d** Real-time qRT-PCR validated miR-9-5p expression in the serum exosomes of MDD patients

The metastasis of exosomal content, particularly miRNAs, was involved in various pathophysiological developmental processes in the process of tumor metastasis [33]. To assess the role of exosomes in MDD pathophysiology, we profiled and analyzed miRNA expression in serum exosomes from HC subjects and MDD patients (Guangzhou Ribo Bio Co., Ltd.) (Fig. 2a). Volcano plot showed 251 significantly differentially expressed miRNAs, including 167 miRNAs with increased expression and 84 miRNAs with decreased expression in MDD patients (Fig. 2b). We verified the expression of the two top upregulated miRNAs [log2 (fold change) ≥ 2]. MiR-9-5p was the only one passed RT-PCR validation (Fig. 2c, d). Thus, exosomal miR-9-5p may serve as a potential messenger in the pathogenesis of MDD and may be developed as a marker for MDD diagnosis.

### PC12 cell-derived exosomal miR-9-5p contributed to microglial M1 polarization

**Fig. 3:**
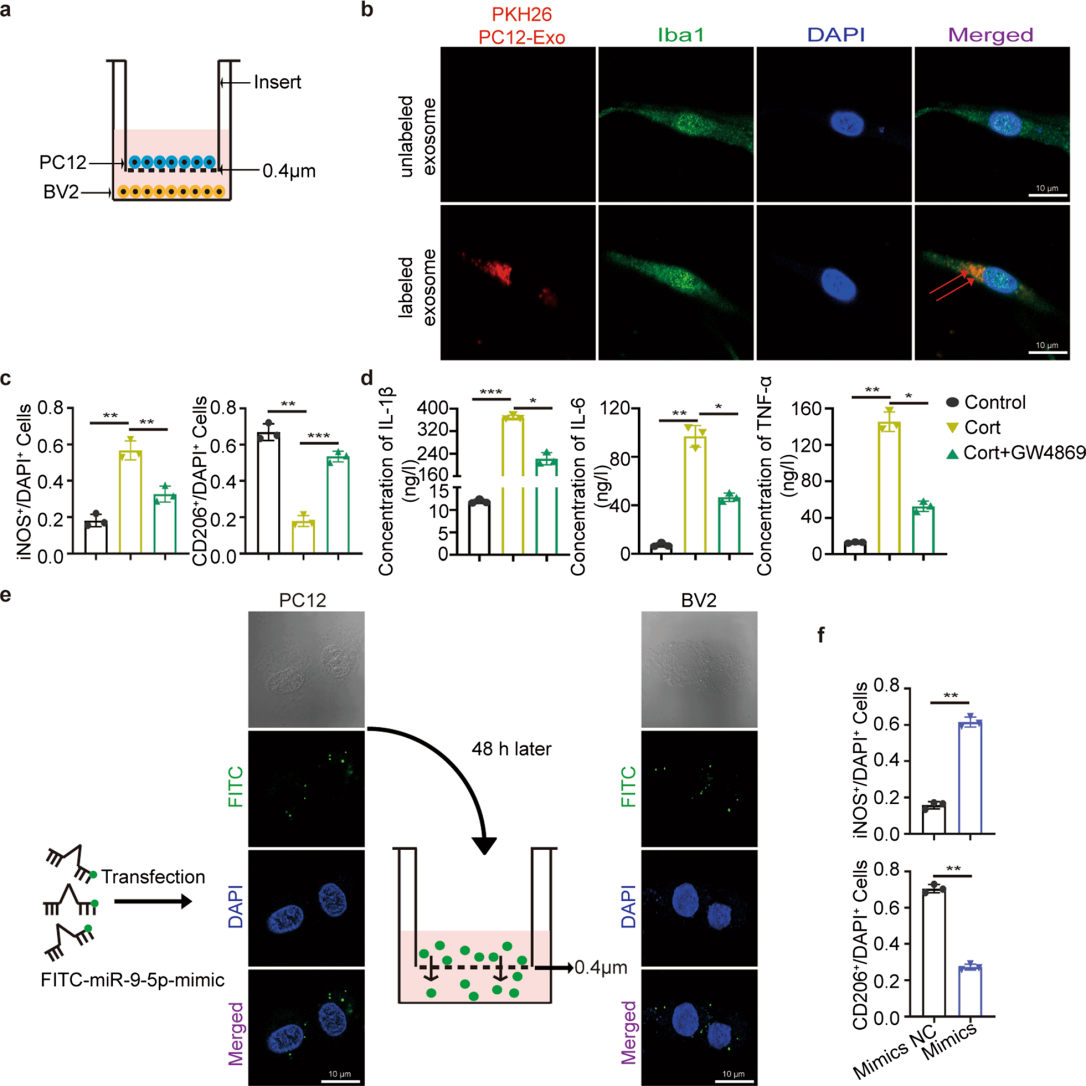
Depression-cells-derived exosomes containing miR-9-5p were engulfed by microglia and promoted the polarization of microglia.** a** Schematic diagram of PC12/BV2 cell co-culture system. **b** Internalization of PKH-26-labeled exosomes derived from PC12 cells was analyzed in BV2 cells. Scale bar = 10 µm. **c** Quantification of iNOS^+^ and CD206^+^ microglia in the three groups. **d** Extracelluler inflammatory cytokines IL-6, IL-1β and TNF-α levels in indicated three groups. **e** PC12 cells transfected with the FITC-miR-9-5p mimic (green fluorescence) were plated in the upper chamber and coincubated with BV2 cells in the lower chamber in a coculture system with a 0.4-µm pore membrane. Green fluorescence was detected in the PC12 recipient cells under the fluorescence microscope. Scale bar = 10 µm. **f** Quantification of iNOS^+^ and CD206^+^ staining in microglia in the two groups

Given that neuronal exosomes might activate microglia [34], we examined PC12 cells derived exosomes in a Transwell coculture system with a 0.4 μm porous membrane (Fig. 3a). PKH26-labeled PC12 exosomes with high miR-9-5p contents were added to the culture medium (Additional file 1: Fig. S3a). As shown, BV2 cells internalized PC12-derived exosomes, leading to increase of miR-9 levels (Fig. 3b). The up-regulation of miR-9-5p was significant in BV2 cells treated with CORT compared to its control (Additional file 1: Fig. S3b). Consistently, the number of M1 microglia was also increased (Fig. 3c). In addition, the intracellular level of the inflammatory factors IL-1β, IL-6 and TNF-α were significantly upregulated in M1 microglia (Fig. 3d). These functions were completely reversed by pretreating PC12 cells with GW4869. We then confirmed that primary neurons secreted miR-9-5p-containing exosomes as well (Additional file 1: Fig. S3c). Both in vitro and in vivo, miR-9-5p levels were much higher in neuronal cells than microglia (Additional file 1: Fig. S3d). When PC12 cells were transfected with FITC-miR-9-5p mimics which confirmed by detection of strong fluorescence (Fig. 3e), an increase of M1 microglia number was observed (Fig. 3f). Taken together, PC12-derived exosomal miR-9-5p promoted M1 microglia polarization.

### Upregulated miR-9-5p in microglia promoted M1 polarization in vitro and in vivo

**Fig. 4:**
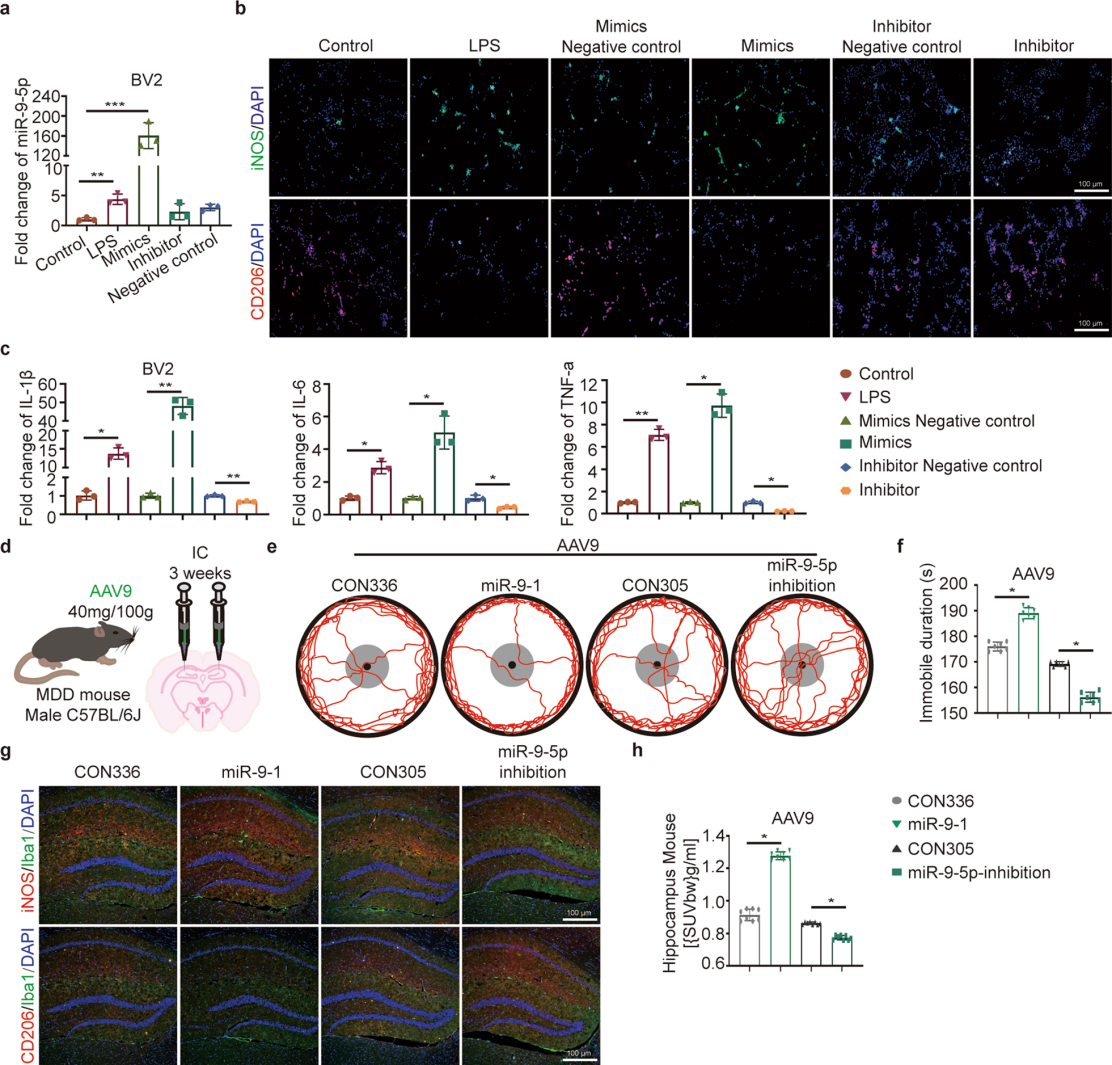
miR-9-5p promoted M1 polarization of microglia in vitro. **a** Related miR-9-5p level in the different groups. **b** iNOS^+^ and CD206^+^ staining for microglia under various treatments. Scale bar = 100 µm. n = 3. **c** Related fold change of IL-1β, IL-6 and TNF-α mRNA. Their relative expression levels were measured by 2^−ΔΔCt^. **d** Schematic diagram of adenovirus stereotactic injection into mouse brain. **e**, **f** Representative tracks and immobile duration of mice in different groups. n = 6. **g** Immunofluorescence for brain tissues in indicated groups. Scale bar = 100 µm. n = 6. **h** [^18^F]DPA-714 uptake in uptake hippocampus

Next, we manipulated the level of miR-9-5p in BV2 cells by inducing overexpression or applying corresponding miRNA inhibitors (Fig. 4a). As expected, in miR-9-5p-overexpressed BV2 cells, the proportion of iNOS^+^ cells was significantly increased (Fig. 4b, Additional file 1: Fig. S4a), and the expression of IL-1β, IL-6, and TNF-α was upregulated (Fig. 4c). We then used the AAV system to deliver miR-9-5p and examine its function in MDD mice (Additional file 1: Fig. S4b,c). An eGFP reporter on viral backbone was used for tracing delivery of virus in vivo. Coronal brain sections were imaged for checking delivery efficiency in the mouse hippocampus after three weeks post stereotactic cerebral injection (Additional file 1: Fig. S4d). Comparing to control group, MDD mice injected with AAV carrying miR-9-1 showed increased depression-like behavior in the OFT and FST (Fig. 4d–f, Additional file 1: Fig. S4e). Moreover, both the M1 microglial number and [^18^F]DPA-714 signals were increased in the hippocampus (Fig. 4g, h, Additional file 1: Fig. S4f), and M1 polarization in primary microglia was also enhanced. (Additional file 1: Fig. S4g). These results indicated that miR-9-5p induced neuroinflammation and exacerbated depression. Contrarily, virus carrying miR-9-5p inhibitors alleviated depression in mice. To sum up, raised miR-9-5p contributed to microglial M1 polarization and neuroinflammation in vivo and in vitro.

### miR-9-5p promoting microglia polarization was mediated by SOCS2-STAT3 axis

**Fig. 5:**
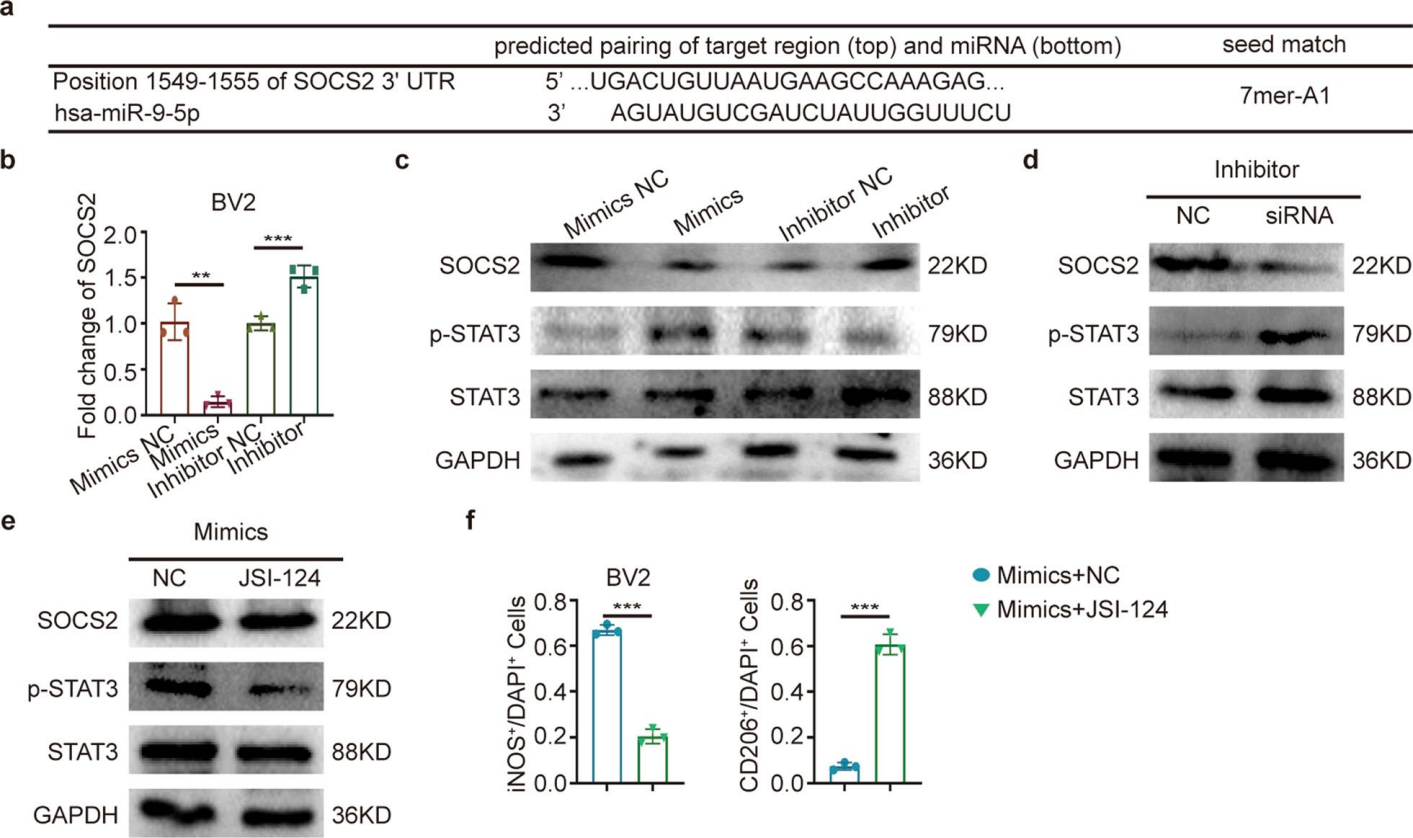
Exosomal miR-9-5p activated STAT3 pathway through suppressing SOCS2.** a** Sequence alignment of miR-9-5p and SOCS2 3′UTR sequences. **b** Real-time qRT-PCR confirmed the effects of miR-9-5p mimics and inhibitor on SOCS2. **c** Protein levels of SOCS2, p-STAT3 and STAT3 in the BV2 cells treated with mimics and inhibitor. n = 3. **d** SiRNA reversed the effect of inhibitor to SOCS2 and p-STAT3. n = 3. **e**, **f** Western blot analysis of SOCS2, p-STAT3 and STAT3 expression and quantification of iNOS^+^ and CD206^+^ staining in microglia treated with mimics and JSI-124. GAPDH served as the loading control. n = 3

To further investigate the underlying mechanism that miR-9-5p promotes microglial polarization, we predicted potential miR-9-5p targets with TargetScan. We identified miR-9-5p binding sites in the 3'-UTR of SOCS2 mRNA (Fig. 5a), which was involved in STAT3 pathway activation and able to mediate microglial polarization regulation [35–37]. Inspired by these findings, we validated whether SOCS2 was a major mediator in miR-9-5p regulating miR-9-5p. As shown in Fig. 5b, c and Additional file 1: Fig. S5a–c, the SOCS2 mRNA and protein levels were attenuated in the mimic group and elevated in the inhibitor group. In addition, siSOCS2 counteracted the miR-9-5p inhibitor’s function on SOCS2 and p-STAT3 activation (Fig. 5d, Additional file 1: Fig. S5d). Moreover, inhibition of the JAK/STAT3 pathway by cucurbitacin I (JSI124) [38] largely rescued p-STAT3 activation and M1 polarization upon miR-9-5p mimic treatment (Fig. 5e, f, Additional file 1: Fig. S5e, f). These data validated that miR-9-5p regulated BV2 cell polarization by targeting the SOCS2/STAT3 signaling pathway.

### Polarized microglia resulted in the accumulation of PC12 cell damages

**Fig. 6:**
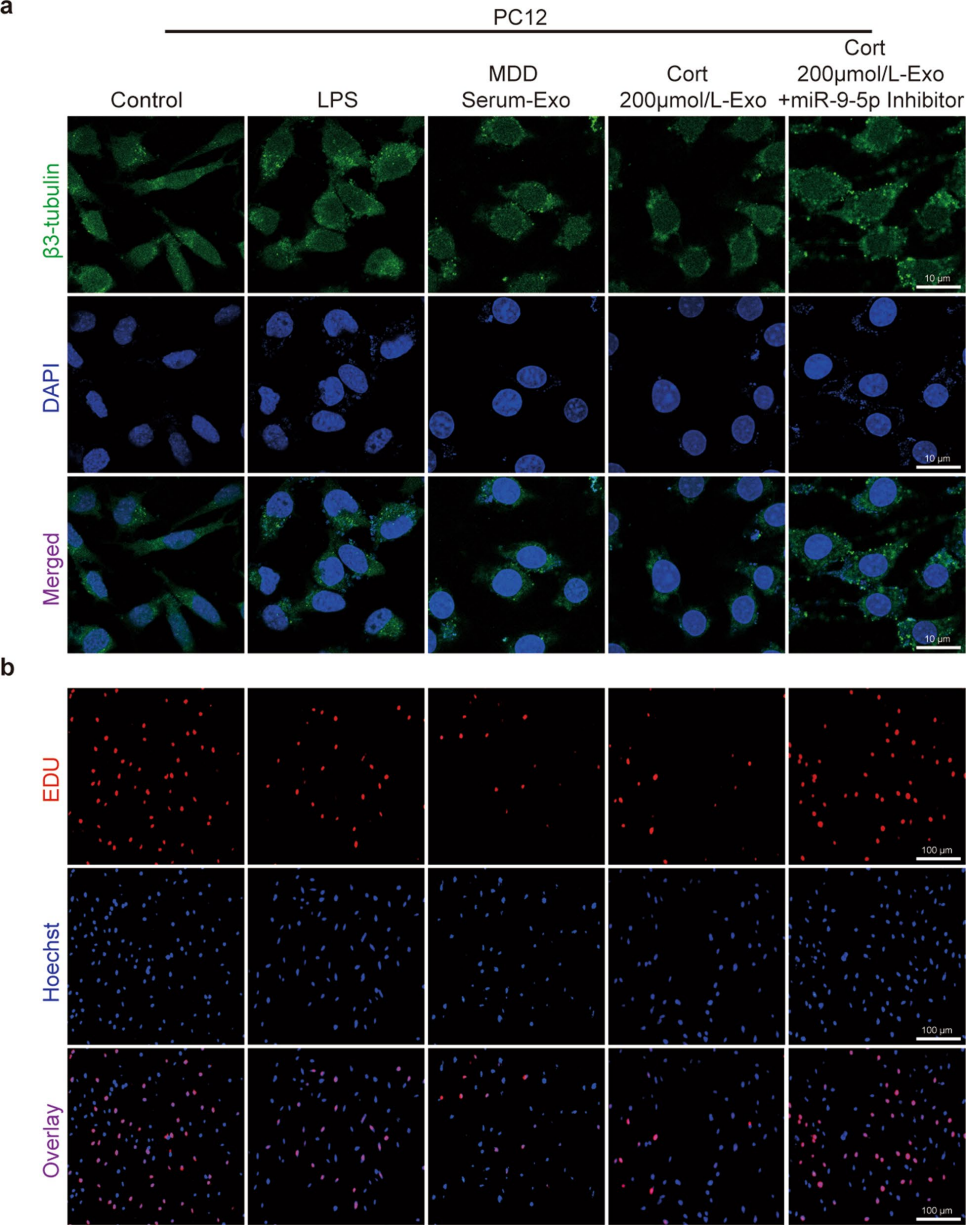
Polarized M1 cells contributed to damaging PC12 cells. To further study the changes in PC12 cells cocultured with M1 microglia, β3-tubulin and EdU immunofluorescence staining were performed to measure synaptic growth and cell proliferation. **a** Total neurite length of PC12 cells after different treatments was imaged by β3-tubulin staining. Scale bar = 10 µm. n = 3. **b** PC12 cell proliferation after co-culture was detected by EdU staining. Scale bar = 100 µm. n = 3

As previously reported, M1 macrophage polarization contributed to inflammation in patients with neurodegenerative disorders exhibiting severe depression [39]. Inflammatory factors, which released by polarized M1 microglia, further aggravated the progression of neuroinflammation and inhibited the neurite outgrowth [22]. To simulate the interaction of the two cells in the brain, transwell systems were used with PC12 planted in the lower chamber and BV2 in the upper chamber (Additional file 1: Fig. S6a). M1 microglia, pretreated with LPS, serum exosomes from MDD patients or exosomes from MDD cellular models, directly shortened neuronal synapses and inhibited cell proliferation when co-cultured with PC12 cells. In contrast, the addition of miR-9-5p inhibitors improved these cellular properties (Fig. 6a, b, Additional file 1: Fig. S6b, c). These results suggested that M1-polarized microglia resulted in the accumulation of PC12 cell damage.

## Discussion

Neuroinflammation is a prominent mechanism in MDD development [40]. The activation of microglia is involved in neuroinflammation, responding to various of insults (i.e., macrophages) occurred within the CNS. Studies elaborated that the basis for the progression of MDD is continuous inflammation induced by macrophage-associated cytokines [19]. Our findings suggested that activated microglia-induced inflammatory factors were delivered to the CNS, stimulating neuronal cells and developing depressive symptoms. In addition, microglia-mediated neuroinflammation exacerbated neuronal damage, and M1 macrophage-derived IL-1β, IL-6 and TNF-α were associated with cognitive and behavioral symptoms in MDD. Profound disturbances in neurotransmitter systems and severe deficits in synaptic plasticity were both strongly associated with neuroinflammation, and this was the key mechanism among identified neurobiological determinants of MDD [41].

Various cell types-derived exosomes influenced neuron-glia communication and the nervous system development in the CNS [42, 43]. A recent study showed that microglial function was modulated by BMSC-derived exosomes in severe neurological disorders, resulting in extreme anti-inflammatory effects [44]. In our work, depressed neuronal exosomes activated microglia and released large amounts of inflammatory factors, causing cumulative inflammatory damage and perturbing the neuronal cell cycle. Bioinformatic prediction also supported that the high enrichment of MDD-affected miRNAs and miRNA modules targets in genes associated with more active neural dendrites, axons and synapse development and transmission [18]. Thus, we aimed to use depressed neuronal cell-derived exosomes, which could deliver miRNAs across the BBB, to target microglial polarization, and assess neuroinflammation development and depression behavioral symptoms exacerbation in mice. Our study confirmed that exosomal miRNAs played a vital role in the pathogenic mechanisms of depression and paved a new avenue for studying MDD development.

MiR-9, which is expressed in the developing embryonic CNS, is highly conserved and facilitates neurogenesis and differentiation [45–48]. Circular RNA DYM (circDYM) levels were significantly decreased both in the peripheral blood of MDD patients and in the CUMS mouse models. The function of CircDYM was acting as an endogenous miR-9 sponge to restrain its activity [49]. Brain miR-9 levels in mood disorder patients was positively correlated to its levels in peripheral blood [50], which is consistent with our observation in serum exosomes from MDD. In our finding, MDD cellular model had significantly higher exosomal miR-9-5p expression levels. LPS-injection also induced miR-9 expression in Iba-1-labeled mouse microglia [51]. In addition, anti-miR-9 microinjection inactivated microglia in the CNS upon LPS stimulation, resulting in alleviated neuroinflammation in mice [52]. Consistent with this finding, we found a strong association between miR-9-5p-induced microglial activation, inflammatory factor releasing and depressive-like behavior progression in mice. The involvement of miRNAs in regulating transcription factors to promote certain pathological processes by SOCS was demonstrated [53]. SOCS is characterized by possessing a central Src homology 2 (SH2) structural domain and a carbon-terminal SOCS box that negatively regulates JAK-STAT signaling [54]. The JAK-STAT pathway is a common signaling cascade in which STAT3 plays a significant role in controlling the inflammatory response, innate immunity and adaptive immunity [55, 56]. In this study, we confirmed that miR-9-5p activated JAK/STAT3 by suppressing SOCS2, thereby inducing M1 polarization. Therefore, the miR-9-5p-SOCS2-STAT3 proliferation axis is involved in the underlying mechanism of microglial polarization, which might add novel evidence for the inflammatory depression hypothesis.

The main limitation of the MDD inflammation hypothesis is lacking measurements of neurological inflammation in MDD patient brain [41]. The pathophysiology of MDD, as an inflammatory CNS disease, can be better understood by PET/CT imaging [57]. [^18^F]DPA-714 was extensively studied in preclinical and clinical models [58–61], as a biomarker of neuroinflammation in various human CNS disorders [58]. To quantify neuroinflammation in patients with depression, we used new radioactive particle tracing technology in PET/CT imaging. However, to date, there was less study using PET/CT imaging to measure neuroinflammatory changes in depression mouse models. Therefore, to better trace the immune status in the mouse brain, we adopted [^18^F]DPA-714 PET/CT imaging as a noninvasive marker for microglial activation. In the present study, we also used this approach to monitor neuroinflammation in the mouse hippocampus. With combination of iNOS antibody staining on brain sections in vitro, and PET/CT imaging in vivo, we confirmed this approach could be applied to assess microglial polarization. Moreover, [^18^F]DPA-714 has the advantages of a high binding affinity and target-to-background ratio [57]. We validated the feasibility to monitor dynamic neuroinflammatory progression in MDD mouse models with [^18^F]DPA-714 PET/CT, and our work could be taken as a successful example.

In this study, we demonstrated that enhancing miRNA-9-5p levels in PC12-derived exosomes can exacerbate cognitive and behavioral impairments in MDD mice, along with neuronal cell damage and neuroinflammation. Our study proposed new ideas for treating depressed neurons by the means of exosomal or miRNA-directed interventions. The model of neuron-microglial communication we used is also a promising and innovative strategy for treating neuroinflammation in MDD. Based on our findings above, there are still some questions that deserve further elucidation. First, our work proposed a novel mechanism to modulate BV2 cell polarization in MDD that could affect the brain-derived inflammatory microenvironment. However, microglial polarization is not linear and static but multidimensional. We evaluated the roles of polarized microglia and found they have similar function on MDD at multiple time points. In future studies, a subtle dynamic balance between M1 and M2 phenotypes in the microenvironment will be studied in depth, to further improve the current research strategy. Second, we identified that polarized microglia caused neuronal damage and that neuronal damage may have also induced microglial inflammation. This potential malignant neuroinflammatory feedback loop warrants further investigation. Finally, our results merit further investigation to explore the ways to ameliorate neuroinflammation through exosomal transport and direct miRNA-related interventions in microglial polarization. Therefore, further research is needed to better develop the idea to manipulate gene regulation by exosome intervention into a precise therapy strategy for more neuroinflammation-related diseases.

## Conclusions

**Fig. 7:**
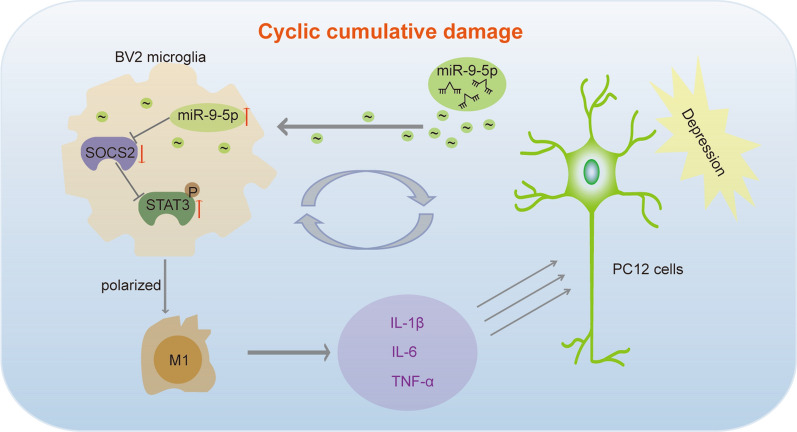
The schematic representation of the interaction between PC12 cells and BV2 microglia in depression. The schematic representation of cyclic cumulative damage: first, the depressed PC12 cells generated exosomes with high miR-9-5p contents. BV2 microglia phagocytosed these exosomes, in which enriched miR-9-5p, suppressed SOCS2 expression and activated JAK/STAT3 pathways, leading to polarization into M1 type. Meanwhile, proinflammatory factors including IL-β, IL-6 and TNF-α were secreted by M1 microglia. These proinflammatory factors further accelerated PC12 cell damage

Collectively, miR-9-5p is a key factor in regulating microglial polarization to promote MDD pathological progress. miR-9-5p was enriched in exosomes derived from depressed neurons, and it strongly promoted inflammatory factors release via suppressing SOCS2 expression and activating the JAK/STAT3 pathways (Fig. 7). miR-9-5p has the potential to be developed as a promising novel therapeutic target for treating neuroinflammatory disorders including MDD.

## Materials and methods

### Subjects and sample preparation

We randomly recruited 3 HC subjects and 6 MDD patients without underlying disease from the Fifth People’s Hospital of Zhenjiang City, China. The Hamilton Depression Rating Scale (HAMD) and Montgomery Asperger's Depression Rating Scale (MADRS) were used to assess the severity of MDD. The study was approved by the Ethics Committee of the Fifth People's Hospital of Zhenjiang, China, and written informed consent was obtained from all participants or their legally authorized representatives. Blood samples were obtained from the subjects, and serum exosomes were isolated for sequencing. Differential miRNA expression analysis was performed, and the data were compared between MDD patients and HC subjects. The original sequencing data are provided in the supplementary materials (Additional file 1: Fig. S2). Detailed demographic information and characteristics of the sequenced patients are shown in Additional file 1: Table S1.

### Cell culture

The PC12 cell line, a commonly used rat neuronal cell line, was acquired from the Central Laboratory of Jiangsu University, Zhenjiang. BV2 cells (i.e., mouse microglia) were purchased from the Cell Bank of the Chinese Academy of Sciences (Shanghai, China). All cells were cultured with high-glucose DMEM containing 10% exosomal-free FBS (Thermo Fisher, Carlsbad, CA), 100 mg/ml streptomycin and 100 U/ml penicillin. The cells were all cultured at 37 °C and 5% CO_2_ in a humid environment.

### Primary neuron isolation and culture

Primary hippocampal neurons were harvested from E18 rat embryos as described in the previous study [62]. The hippocampus was isolated from the brain and cut into small 1 mm^3^ pieces in phosphate-buffered saline (PBS, pH 7.4). Minced tissue was further digested with 0.125% trypsin solution at 37 °C for 20 min, and then stopped by DMEM/F12 medium with 10% FBS. The digested tissue was filtered with a 200-mesh filter cloth. After brief centrifugation, the cells were resuspended in DMEM/F12 containing 20% FBS and 1% penicillin/streptomycin, seeded in 6-well plates precoated with poly-L-lysine and incubated at 37 °C, 5% CO_2_ and 95% O_2_ for 24 h. To selectively isolate neurons, the medium was replaced with culture medium consisting of Neurobasal-R medium, 2% B27, 0.5 mmol/l L-glutamine and 1% penicillin–streptomycin. Medium was replaced once every 2 days. Approximately 6–8 days later, the cultured cells were ready for subsequent experiments.

### Primary microglia isolation and culture

Cortical tissues were obtained from P0-2 C57BL/6 mouse pups, stripped of the meninges, and mechanically dissociated with a hand homogenizer and a 25-gauge needle. The cell suspensions were seeded into poly-L-lysine (Sigma–Aldrich)-coated T150 tissue culture flasks and maintained in DMEM/F12 containing 10% FBS and 1% penicillin–streptomycin for 10–14 days to obtain a confluent mixed astrocyte/microglia population. Microglia were isolated from the mixed glial cultures by shaking on an orbital shaker at 220 rpm for 1 h. The supernatant, which contained detached microglial cells, was collected and reseeded for 1 h to allow microglial attachment. After 1 h, the remaining nonadherent cells were removed.

### Exosome isolation, quantification and labeling

The details are provided in Additional file 1: Supplementary methods.

### RNA extraction and qRT-PCR

The details are provided in Additional file 1: Supplementary methods.

### Transfection

The details are provided in Additional file 1: Supplementary methods.

### Transwell assay

The details are provided in Additional file 1: Supplementary methods.

### Western blot analysis

The details are provided in Additional file 1: Supplementary methods.

### ELISA

The details are provided in Additional file 1: Supplementary methods.

### Immunofluorescence

The details are provided in Additional file 1: Supplementary methods.

### Animals and establishment of an animal model of depression

All animal experiment protocols were approved by the Institutional Animal Care and Use Committee (IACUC) of the Medical School of Jiangsu University, according to the laboratory guidelines for the ethical review of animal welfare. Male C57BL/6 J mice (25–27 g, 8–10 weeks old) were purchased from Changzhou Cavens Experimental Animal Center (animal certification number: SCXK (SU) 2016-0010) and adapted to the environment for 7 days before the experiments. An animal model of CUMS was chosen in our study. The procedures included (1) short-term cage feeding (5 animals per cage) for 3 h; (2) exposure to fear-inducing odors (rats' padding) for 3 h; (3) exposure to noise (an unmodulated radio) for 3 h; (4) 30° cage tilt overnight; (5) exposure to wet bedding (150 ml water) overnight; (6) exposure to a new cage without bedding overnight; and (7) exposure to light overnight.

### MiR-9-5p inhibitor virus

Production and packaging of adeno-associated virus particles: the exogenous gene is first cloned into the viral vector, then the packaging plasmid is transfected in 293 cells, then the AAV virus particles are collected, concentrated and purified, and finally the titer of the resulting virus is determined by quantitative PCR. The miR-9-5p precursor sequence and sequences extending upstream and downstream by about 100 bp each or a polyclonal site using a generic flank sequence as an extension are constructed on the vector. Antisense sequences expressing the miR-9-5p mature through a type II or type III promoter are the construction option for the miR-9-5p inhibitor virus (GENEchem, Shanghai, China). The principle of miR-9-5p down is to reduce the inhibition of target gene mRNA translation by the antisense miR-9-5p sequence by competing for binding to the intracellular miR-9-5p mature, thereby affecting the binding of the miR-9-5p mature to the target gene mRNA. Locations and sequences of sequencing primer: pGCSIL-F (4185- 4165): CCATGATTCCTTCATTTGC. The sequencing sequence of the miR-9-5p suppressor virus is as follows: ACCGTCATACAGCTAGATAACCAAAGATTTTT. The titer of the miR-9-5p inhibitor virus is 3.21 × 10^12^ v.g./ml.

### Stereotactic cerebral injection

The AAV virus used for stereotaxic injections was obtained from GENEchem (Shanghai, China). A description of the miR-9-5p inhibitor virus is provided in Additional file 1: supplementary information. The mice were anesthetized by intraperitoneal injection of chloral hydrate (40 mg/100 g). Then, head skin was cleaned and disinfected, and the whole body was fixed to a stereotaxic instrument. The injection coordinates for the hippocampus were determined (1.7 mm posterior and 1.8 mm mediolateral to the anterior fontanelle and 2.0 mm deep), a cranial window was made, and a microinjection needle was fixed in place. The virus was injected into 1 site per hemisphere; the injection volume was 1 µl, and the injection rate was 0.2 µl/min.

### Behavioral assessment

OFT. MouseLabTracker software was used to track the movement of the mice during the habituation phase of the object displacement task, which was similar as open field exploration task. The center of the camera view circle was aligned to the center of the arena, and the radius of the circle was one-third of the radius of the arena (15 cm). Further data analysis was carried out by calculating the path traveled and the time spent in the central area.

FST. The mice were placed in a cylindrical glass swimming tank with a height of 20 cm, a diameter of 12 cm, a depth of 10 cm, and a water temperature of 25 °C. The mice were monitored for 6 min, and the immobility time within 4 min was recorded (the immobility standard was that the body was in a floating state or only the limbs moved slightly to ensure that the head was floating on the water).

### PET/CT imaging

Micro-PET/CT was used for dynamic body scan for mice. The PET imaging system was an Inveon small animal PET/CT system (Siemens Preclinical Solution). The mice were weighed, anesthetized with isoflurane (the induction concentration was 3%, the maintenance concentration was 2–2.5%) in air, fixed on the scanning bed, and injected with [^18^F]DPA-714 (0.2 ml/100 g, 3.7–5.5 mBq) through the tail vein for 1 h. The scanning parameters were: layer thickness 1 mm, matrix 128 × 128, current 500 μA, voltage 80 kV, and acquisition energy window 350–650 kV. The dynamic data collected by micro-PET were reconstructed by the following frame segmentation methods: 6 × 10 s, 4 × 60 s, 5 × 300 s, and 3 × 600 s. Based on the maximum a posteriori (MAP) algorithm, three-dimensional ordered subset expectation maximization (3D-OSEM) was used to reconstruct the data images of each frame. The reconstructed micro-PET images were observed with Inveon Research Workplace (IRW 3.0) software and used for region of interest (ROI) analysis, and the ROIs of the brain organs and tissues were drawn on the images. Finally, the time-radio activity curves (TACs) of the ROIs at different time points (5 s, 15 s, 25 s, 35 s, 45 s, 55 s, 90 s, 150 s 210 s 270 s 450 s 750 s 1050 s, 1350 s, 1650 s, 2100s, 2700 s and 3300 s) were obtained to display the dynamic characteristics of the imaging agent in the brain, major organs and tissues. The percentage injected dose per gram (% ID/g) of tissue is expressed as the value of imaging agent injected, which is calculated by assuming the organ tissue density to be 1 g/ml and dividing the radioactive activity value of each organ tissue by the total injected dose to obtain the % ID/g value.

### NIRFI

After purification, exosomes in indicated different densities were added to PBS with 1 mM DiR and incubated at room temperature (RT) for 30 min. The labeled exosomes were then separated from the unbound DiR by two rounds of ultracentrifugation. Images of the DiR-labeled exosomes were taken with an IVIS spectrum imager. On day 0, images of the mice were captured as a baseline. Then, the mice were injected with DiR-labeled and unlabeled exosomes (100 µg, 200 µl PBS) through the tail vein, and then anesthetized with pentobarbital and maintained in the prone position. Similarly, images were captured at 1, 3, 5, 7, 10 and 14 days. On the 14th day, the mice were sacrificed, and the brain, heart, lung, intestine, kidney, spleen and liver were extracted for fluorescence imaging. The fluorescence signals in the tissues were quantified in IVIS software.

### Statistical analysis

Statistical analyses were carried out with GraphPad software. In our study, at least three biological replicates were performed for each experiment, with data expressed as the mean ± SD. Student’s t-test was used to assess the statistical significance between the two groups. Differences between two or more groups were assessed using one-way ANOVA. *p < 0.05, **p < 0.01, and ***p < 0.001.

## Supplementary Information

**Additional file 1: Table S1.** Characteristics of HC and MDD patients. **Figure S1. a** Identification of BV2 cells by Iba1 and their morphological changes after LPS treatment under fluorescence microscopy. Scale bar = 50 μm. **b** Quantification of iNOS^+^ and CD206^+^ staining in microglia in the four groups. **c** (Above) schematic diagram of coculture of CORT and PC12 cells to establish a cellular model of MDD. (Below) the changes of PC12 cell proliferation rate after treatment with CORT. **d** (Above) NIRFI of DiR-labeled exosomes after ultracentrifugation from free DiR solution. (Below) linear correlation between the fluorescence signal intensity and exosomes concentration. **e** Representative NIRF images of brains from the DiR-labeled group exosomes-treated and unlabeled exosomes-treated group. **f **(Left) the average radiant efficiency based on NIRF images of isolated organs from the DiR-labeled exosomes-treated group on day 1. (Right) representative NIRF image of tissues from the DiR-labeled exosomes-treated group on days 1 and 14. **g** Representative immunofluorescence images of brains from the PKH26-labeled exosomes-treated group and unlabeled exosomes-treated group. Scale bar = 100 μm.** h** Quantification of behavior during open-field exploration. (Left) Time spent in the center area. (Middle) Percentage path length travelled in the center area. (Right) Total path length travelled. **Figure S2. **Sequencing heat map of miRNAs in serum exosomes of HC subjects and those of MDD patients. **Figure S3.** **a** Real-time qRT-PCR results showed that the expression of miR-9-5p in CORT-treated PC12 cells and their derived exosomes. **b** The changes of miR-9-5p expression in microglia after co-culture with different treated PC12 cells for 48 h were measured. **c** (Left) morphology of primary neuron. Scale bar = 50 μm. (Right) The PCR results showed that primary neuron like PC12 cells were able to secrete exosome containing miR-9-5p. **d** (Left) Iba-1 immunostaining of primary microglia. Scale bar = 50 μm. (Right) the levels of miR-9-5p in neurons and their derived exosomes were significantly higher than in microglia, confirming that the main source of miR-9-5p are neurons. **Figure S4. a** Quantification of iNOS^+^ and CD206^+^ staining in microglia treated as above. **b** Quantification of behavior during field exploration in control group and MDD group. (Left) time spent in the center area. (Middle) percentage path length travelled in the center area. (Right) total path length travelled. **c** the forced swimming time of mice in control group and MDD group. **d** the mouse hippocampus showed the presence of eGFP in AAV9 vectors. Scale bar = 200 μm. **e** The behavior of different adenovirus groups during field exploration was quantified. **f** Quantification of iNOS^+^ and CD206^+^ staining in microglia cells in the hippocampus of mice treated as above.** g** iNOS^+^ and CD206^+^ staining for primary microglia in the two groups were detected. Scale bar = 100 µm. **Figure S5. a**, **b** Real-time qRT-PCR and western blot analysis confirmed the knockdown performance of siSOCS2. **c** Quantification for the expression of SOCS2 or p-STAT3 in microglia treated with mimics or inhibitor. **d** Quantification for the expression of SOCS2 or p-STAT3 in microglia treated with inhibitor and siRNA. **e** Western blot analysis was used to determine the expression of p-STAT3 protein in BV2 cells after JSI-124 (200 nM) for 48 h. **f** Quantification for the expression of SOCS2 or p-STAT3 in microglia treated with JSI-124 and mimics. **Figure S6.** **a** Schematic diagram of co-culture. BV2 microglia cells were implanted into the upper compartment and treated with different treatments. **b**, **c **Quantitative staining of β3-tubulin and EdU in PC12 cells co-cultured for 48h with BV2 cells treated as above.

## Data Availability

All data generated or analyzed during this study are included in this published article.

## Abbreviations

MDD: Depression
HC: Healthy control
Exos: Exosomes
EVs: Extracellular vehicles
miRNAs: Micro-RNAs
IL-1β: Interleukin-1β
IL-6: Interleukin-6
TNF-α: Tumor necrosis factor-alpha
CNS: Central nervous system
NSC: Neural stem cell
LPS: Lipopolysaccharide
CORT: Corticosterone (CORT)
SOCS2: Suppressor of cytokine signaling 2
JAK: Janus kinase
STAT3: Signal transducer and activator of transcription 3
TEM: Transmission electron microscope
HAMD: Hamilton Depression Rating Scale
MADRS: Montgomery Asperger’s Depression Rating Scale
OFT: Open field exploration
FST: Forced swimming experiment
NIRFI: Near-Infrared Fluorescence Imaging
qRT-PCR: Quantitative real-time PCR
SH2: Src homology 2
