# Supplementary material for: Neuron secrete exosomes containing miR-9-5p to promote polarization of M1 microglia in depression
Source: J Nanobiotechnology. 2022 Mar 9;20:122. doi: 10.1186/s12951-022-01332-w (PMC8905830; doi:10.1186/s12951-022-01332-w)
Supplement: Supplementary file 1 — Additional file 1: Table S1. Characteristics of HC and MDD patients. Figure S1. a Identification of BV2 cells by Iba1 and their morphological changes after LPS treatment under fluorescence microscopy. Scale bar = 50 μm. b Quantification of iNOS+ and CD206+ staining in microglia in the four groups. c (Above) schematic diagram of coculture of CORT and PC12 cells to establish a cellular model of MDD. (Below) the changes of PC12 cell proliferation rate after treatment with CORT. d (Above) NIRFI of DiR-labeled exosomes after ultracentrifugation from free DiR solution. (Below) linear correlation between the fluorescence signal intensity and exosomes concentration. e Representative NIRF images of brains from the DiR-labeled group exosomes-treated and unlabeled exosomes-treated group. f (Left) the average radiant efficiency based on NIRF images of isolated organs from the DiR-labeled exosomes-treated group on day 1. (Right) representative NIRF image of tissues from the DiR-labeled exosomes-treated group on days 1 and 14. g Representative immunofluorescence images of brains from the PKH26-labeled exosomes-treated group and unlabeled exosomes-treated group. Scale bar = 100 μm. h Quantification of behavior during open-field exploration. (Left) Time spent in the center area. (Middle) Percentage path length travelled in the center area. (Right) Total path length travelled. Figure S2. Sequencing heat map of miRNAs in serum exosomes of HC subjects and those of MDD patients. Figure S3. a Real-time qRT-PCR results showed that the expression of miR-9-5p in CORT-treated PC12 cells and their derived exosomes. b The changes of miR-9-5p expression in microglia after co-culture with different treated PC12 cells for 48 h were measured. c (Left) morphology of primary neuron. Scale bar = 50 μm. (Right) The PCR results showed that primary neuron like PC12 cells were able to secrete exosome containing miR-9-5p. d (Left) Iba-1 immunostaining of primary microglia. Scale bar = 50 μm. (Right) the [file 12951_2022_1332_MOESM1_ESM.docx]

**Supplementary methods**

Exosome isolation, quantification and labeling

Exosomes in the supernatant of PC12 cells or serum of patients were collected by hypercentrifugation. PC12 cells were cultured in serum without exosomes for 48 h. The collected supernatant or serum was centrifuged at 300×g at 4°C for 10 min, at 2000×g at 4°C for 10 min, and at 10,000 ×g for 30 min to remove cell debris, and then at 100,000 ×g at 4°C for 2h.

For the identification of exosomes, the morphology of the extracted particles was observed using a TEM (Hitachi, Tokyo, Japan) according to the manufacturer's instructions. Briefly, the precipitate was diluted in distilled water (1 mg / ml) and mixed with the same amount of 4% paraformaldehyde. Twenty microliters of the sample was applied to a glow-discharged carbon coated formvar film that was attached to a metal sample grid. The grid was incubated with 50 ml of 1% glutaraldehyde for 5 minutes at room temperature and 8 times with 2 ml of distilled water (2 minutes each). After drying with filter paper for 30 minutes, an equal volume of 10% uranyl acetate was added to the grid for 5 minutes at room temperature, then 50 ml of methylcellulose-uranyl acetate (5 ml of 4% uranyl acetate and 45 ml) 2% was added. Methylcellulose was kept at 4 ° C for 10 minutes. After blotting the excess solution, the sample was dried and examined by TEM. The size distribution of the particles in the particles was measured and analyzed using NTA (Particle Metrix, Meerbusch, Germany). Further, Western blot analysis was first used to detect biomarkers of exosomes including CD9 and CD63. And GW4869 was used as a negative control. Exosomes were placed in a transparent supercentrifuge tube (Beckman Coulter, Indianapolis, USA). Exosomes could be labeled with PKH26 (Sigma-Aldrich, St. Louis, Mo, USA) and DiR (D12731, Invitrogen, Life Technologies). We collected conditioned medium (CM), centrifuged it at 300 g for 5 minutes to remove cells, centrifuged it at 1,500 g for 10 minutes to remove cell debris and then filtered it through a 0.22-mm filter to remove larger particles. The CM was then ultracentrifuged at 110,000 g for 70 minutes to pellet the EVs. A second wash step was performed by resuspending the EV pellet in 25 ml of phosphate-buffered saline (PBS) and ultracentrifuging it at 110,000 g for another 70 minutes. The pellet was then resuspended in PBS. All ultracentrifugation (UC) steps were performed using a Beckman Coulter Type 70 Ti rotor (Brea, CA, USA) at 48°C. The filtered CM was incubated with 1 mM fluorescent lipophilic tracer DiR at room temperature (RT) for 15 minutes prior to EV isolation by the UC method as described above. Leica TCS SP5 II laser scanning confocal microscopy was used to detect the uptake of PKH26-labeled exosomes by BV2 cells. GW4869 (Sigma-Aldrich, Mo, USA) is currently the most commonly used drug to interfere with exosome secretion, blocking exosome formation by inhibiting nSMase2 activity of 2.5 μM.

RNA extraction and qRT-PCR

Total RNA was extracted from cells or human blood exosomes using Trizol reagent (Invitrogen, CA, USA) and converted into cDNA using a reverse transcription kit. All rapid quantitative polymerase chain reactions are performed using the Photocyclometer 96 real-time polymerase chain reaction detection system (Roche Basel, Switzerland). U6 or GAPDH was used as the relative expression level, and the relative level of ribonucleic acid was measured by 2^-ΔΔCt^.

Transfection

At 100 nM, BV2 cells were transfected with Lipofectamine 2000 into miR-9-5p mimics negative control, miR-9-5p mimics, miR-9-5p inhibitor negative control and miR-9-5p inhibitor or siRNA (GenePharma, Shanghai, China), respectively.

Transwell assay

Cells can exchange substances without contact, so a 0.4μm chamber cross-hole system (Corning Corporation, USA) was used to study the effects of corticosterone-treated PC12 cells on BV2 cells and the communication between PC12 cells and BV2 cells. According to the experimental requirements, BV2 cells and PC12 cells were cultured with FBS without exosomes. The primary microglia were stimulated with LPS (1 μg/mL) for 24 h to induce a proinflammatory phenotype. After 48 hours of co-culture, the polarization of BV2 was observed by iNOS and CD206 immunofluorescence staining, respectively or the lower layer of PC12 cells was used for β3-Tubulin fluorescence staining to detect the total length of synapses and EDU staining to detect cell proliferation ability.

Western blot analysis

SDS-polyacrylamide gel was used to detect SOS2 and STAT3 (1:2000; Abcam, Cambridge, MA) and p-STAT3(1:1000; Cell Signaling Technology, USA). GAPDH (1:5000; Abcam, Cambridge, MA) as endogenous controls for protein imprinting analysis. The target protein bands were visualized by Odyssey infrared imaging system.

ELISA

According to the manufacturers’ protocols, the collected cell supernatants were analyzed using ELISA kits to measure the concentration of IL-1β, IL-6 and TNF-α.

Immunofluorescence

Cells were fixed with 4% PFA at room temperature for 15 min, and then treated with 3%BSA at 37℃ for 30 min to block nonspecific staining. They were incubated overnight with primary antibodies, Iba1 (1:500; Abcam, UK), iNOS (1:50; Abcam, UK), CD206 (1:50; Abcam, UK) and β3-Tubulin (1:400; Cell Signaling Technology, USA) at 4℃. Overnight, rinse with PBS for 3x3min, and then incubate at room temperature with secondary antibody for 2h. The nuclei were restained with DAPI. After completion of behavioral testing and PET/CT scanning, mice were deeply anesthetized using isoflurane and euthanized via intercardiac perfusion using 4% paraformaldehyde (Sigma) in 100 mM phosphate buffered saline (PBS, 100 mM, pH 7.4; Gibco). Brains were cryoprotected using a sucrose gradient (10-30%, Sigma) and sectioned coronally into 30μm thick sections using a cryostat (Microm HN 525 NX, Thermo-Scientific, US). Brain tissues sections were incubated with primary antibodies Iba1, iNOS and CD206 at 4℃ for 12 h in a humid environment. The sections were then incubated with a biotinylated secondary antibody for 1h at 37 °C. Tissues were then DAPI nuclear counterstained.

EdU

According to the instructions of the kit, add 50 μmol/L EdU working solution and incubate at 37 ℃ for 2h, then remove the medium. 4% paraformaldehyde was fixed for 30 minutes, and the fixed solution was removed. The cells were incubated with 0.5%TritonX-100 solution decolorization shaker for 10 minutes. The cells were washed with PBS, and the Apollo staining solution was incubated with 0.5%TritonX-100 solution decolorization shaker at room temperature for 30 minutes. The staining reaction solution was discarded, and the reaction solution was incubated with Hoechst 33342 solution at room temperature for 30 minutes, and then washed with PBS.

**Table**

Table S1. Characteristics of HC and MDD patients

| Characteristics | Age(years) | Gender | Classification of depression | HAMD |
| --- | --- | --- | --- | --- |
| MDD1 | 53 | female | Major depression | 37 |
| MDD2 | 17 | female | Major depression | 40 |
| MDD3 | 46 | female | Major depression | 47 |
| MDD4 | 17 | female | Major depression | 36 |
| MDD5 | 62 | female | Major depression | 40 |
| MDD6 | 55 | female | Major depression | 39 |
| HC1 | 25 | male | - | - |
| HC2 | 61 | male | - | - |
| HC3 | 39 | female | - | - |

**Figure**


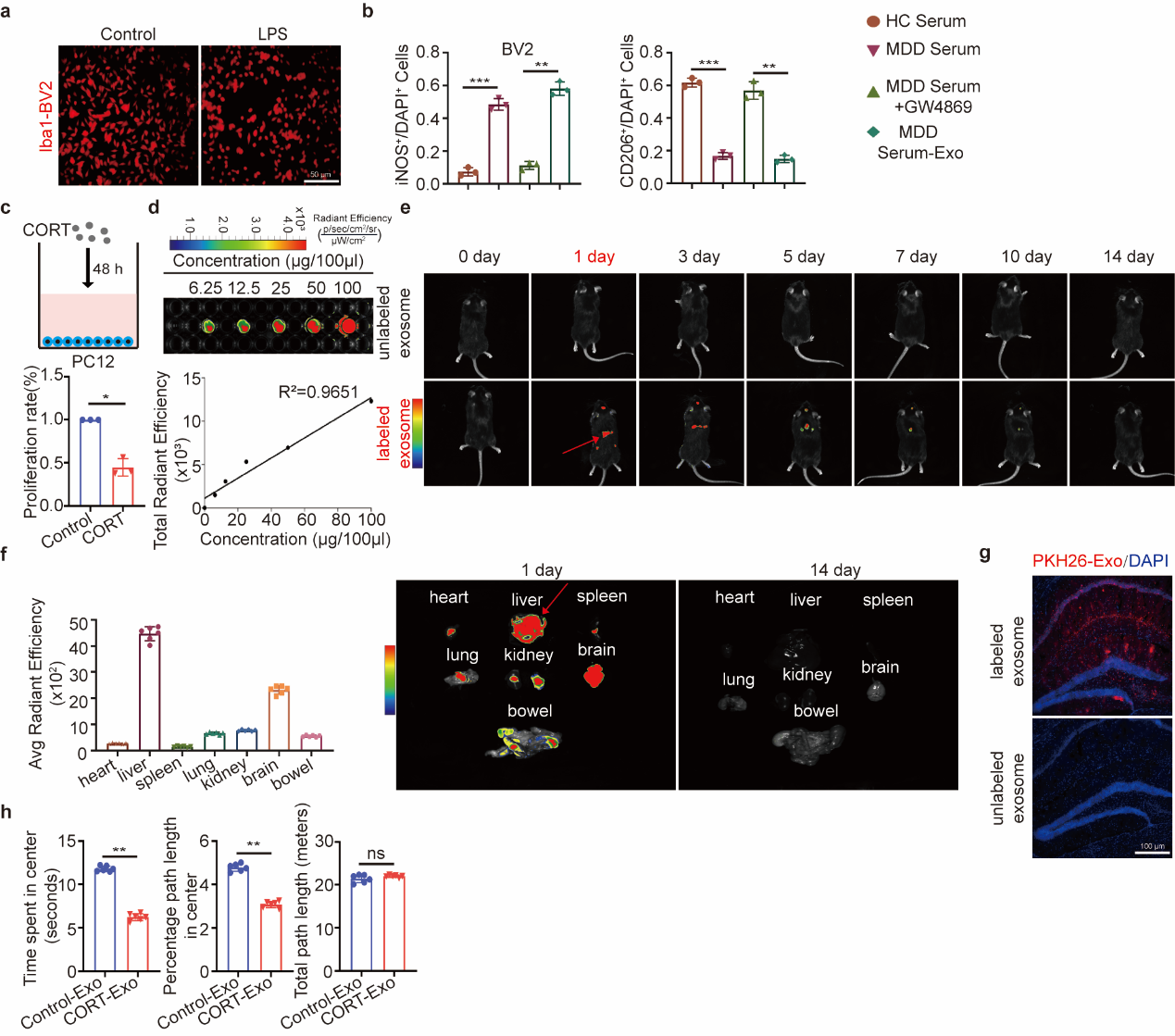


**Fig.S1 a** Identification of BV2 cells by Iba1 and their morphological changes after LPS treatment under fluorescence microscopy. Scale bar=50 μm. **b** Quantification of iNOS^+^ and CD206^+^ staining in microglia in the four groups. **c** (Above) Schematic diagram of coculture of CORT and PC12 cells to establish a cellular model of MDD. (Below) The changes of PC12 cell proliferation rate after treatment with CORT. **d** (Above) NIRFI of DiR-labeled exosomes after ultracentrifugation from free DiR solution. (Below) Linear correlation between the fluorescence signal intensity and exosomes concentration. **e** Representative NIRF images of brains from the DiR-labeled group exosomes-treated and unlabeled exosomes-treated group. **f** (Left) The average radiant efficiency based on NIRF images of isolated organs from the DiR-labeled exosomes-treated group on day 1. (Right) Representative NIRF image of tissues from the DiR-labeled exosomes-treated group on days 1 and 14. **g** Representative immunofluorescence images of brains from the PKH26-labeled exosomes-treated group and unlabeled exosomes-treated group. Scale bar=100 μm. **h** Quantification of behavior during open-field exploration. (Left) Time spent in the center area. (Middle) Percentage path length travelled in the center area. (Right) Total path length travelled.


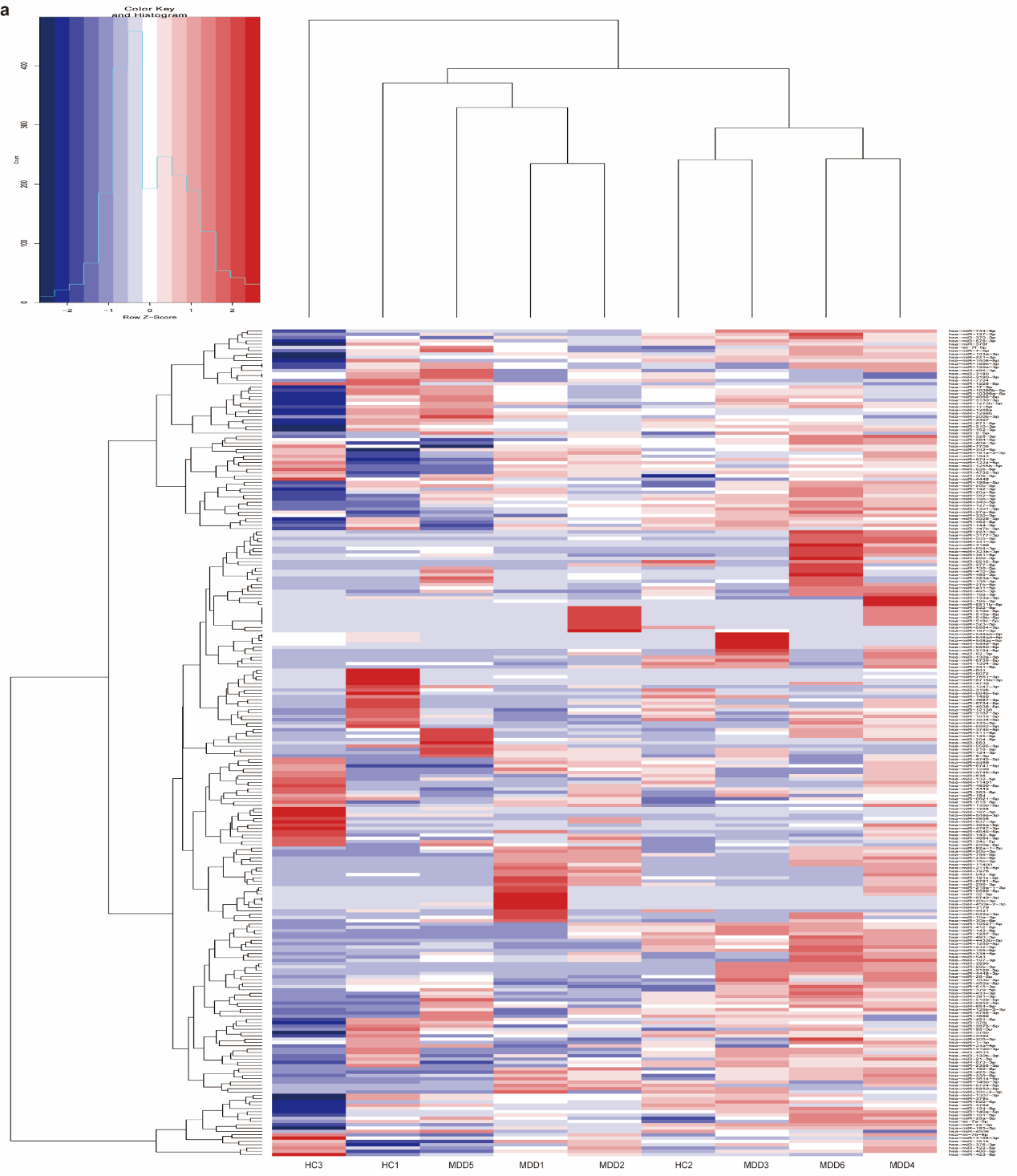


**Fig.S2** Sequencing heat map of miRNAs in serum exosomes of HC subjects and those of MDD patients.


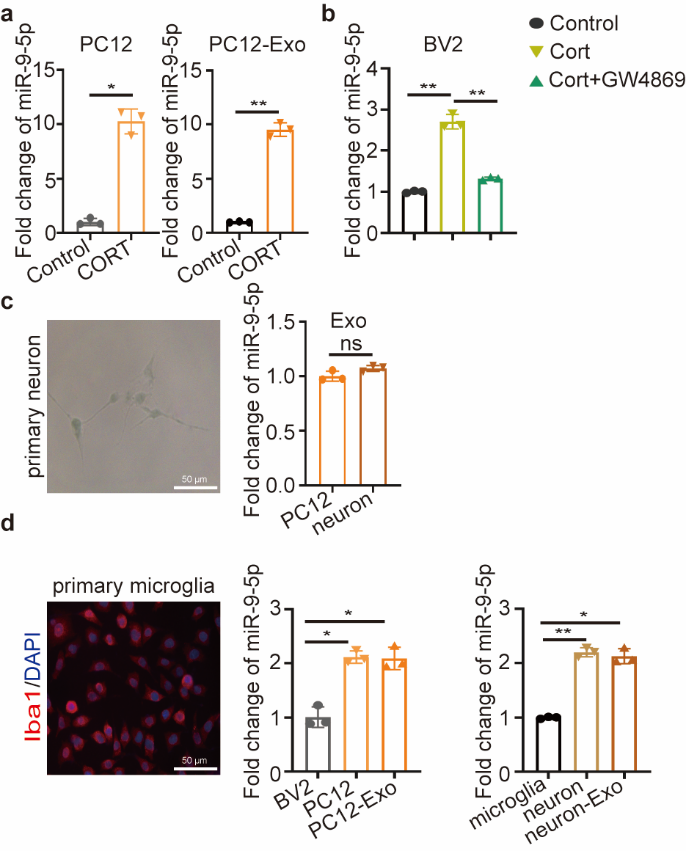


**Fig.S3** **a** Real-time qRT-PCR results showed that the expression of miR-9-5p in CORT-treated PC12 cells and their derived exosomes. **b** The changes of miR-9-5p expression in microglia after co-culture with different treated PC12 cells for 48h were measured. **c** (Left) Morphology of primary neuron. Scale bar=50 μm. (Right) The PCR results showed that primary neuron like PC12 cells were able to secrete exosome containing miR-9-5p. **d** (Left) Iba-1 immunostaining of primary microglia. Scale bar=50 μm. (Right) The levels of miR-9-5p in neurons and their derived exosomes were significantly higher than in microglia, confirming that the main source of miR-9-5p are neurons.


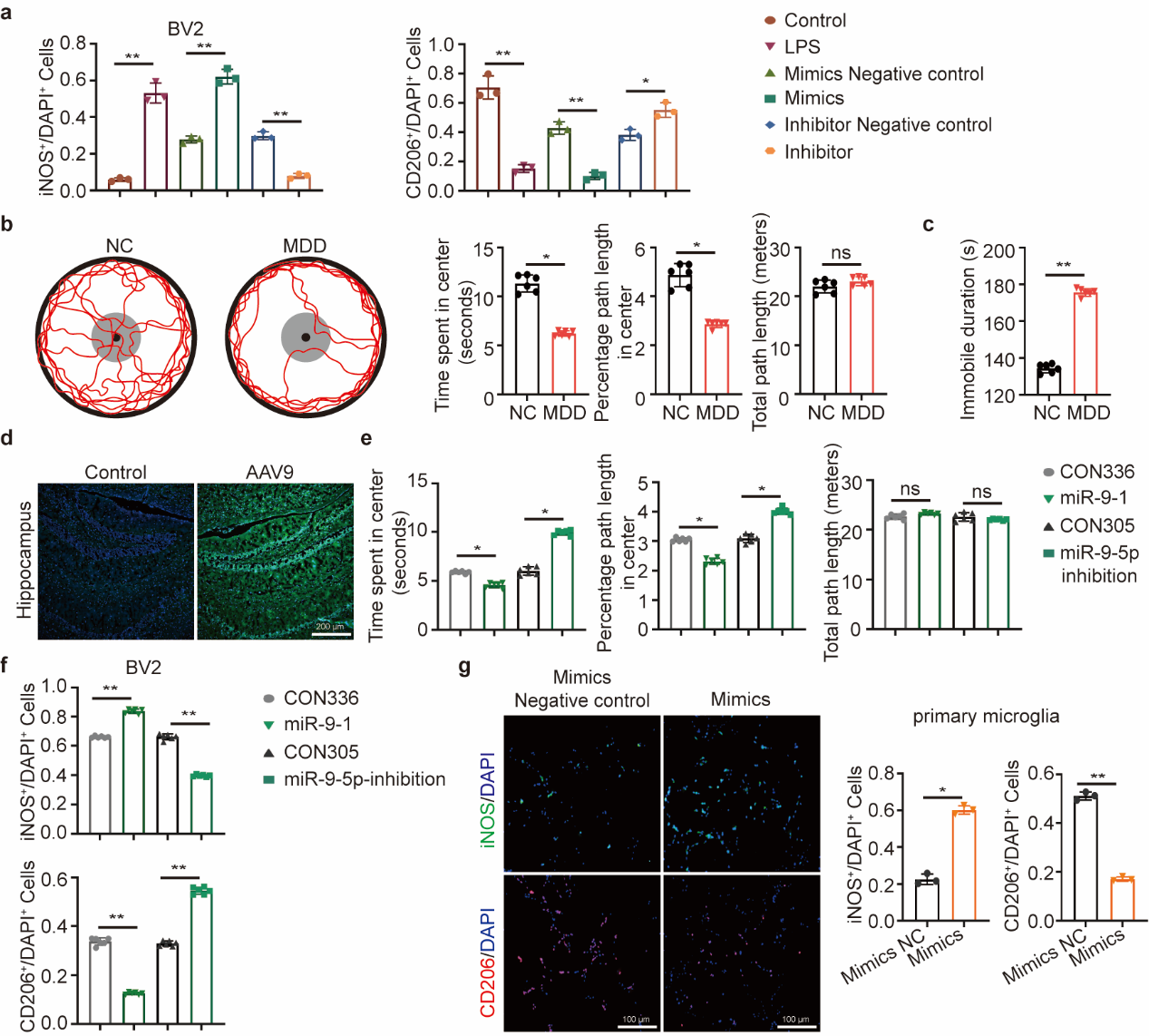


**Fig.S4 a** Quantification of iNOS^+^ and CD206^+^ staining in microglia treated as above. **b** Quantification of behavior during field exploration in control group and MDD group. (Left) Time spent in the center area. (Middle) Percentage path length travelled in the center area. (Right) Total path length travelled. **c** The forced swimming time of mice in control group and MDD group. **d** The mouse hippocampus showed the presence of eGFP in AAV9 vectors. Scale bar=200 μm. **e** The behavior of different adenovirus groups during field exploration was quantified. **f** Quantification of iNOS^+^ and CD206^+^ staining in microglia cells in the hippocampus of mice treated as above. **g** iNOS^+^ and CD206^+^ staining for primary microglia in the two groups were detected. Scale bar=100 µm.


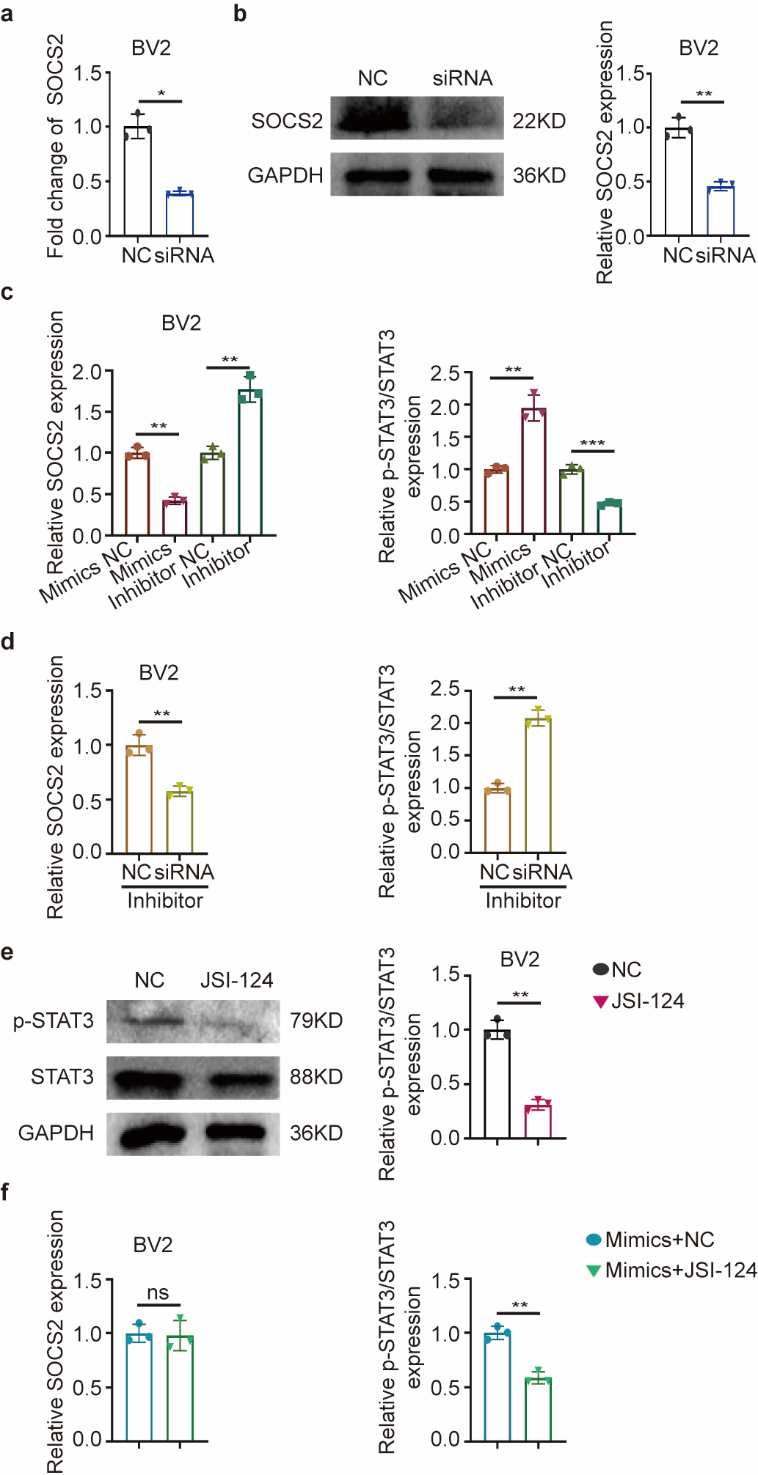


**Fig.S5 a**,**b** Real-time qRT-PCR and western blot analysis confirmed the knockdown performance of siSOCS2. **c** Quantification for the expression of SOCS2 or p-STAT3 in microglia treated with mimics or inhibitor. **d** Quantification for the expression of SOCS2 or p-STAT3 in microglia treated with inhibitor and siRNA. **e** Western blot analysis was used to determine the expression of p-STAT3 protein in BV2 cells after JSI-124 (200 nM) for 48h. **f** Quantification for the expression of SOCS2 or p-STAT3 in microglia treated with JSI-124 and mimics.


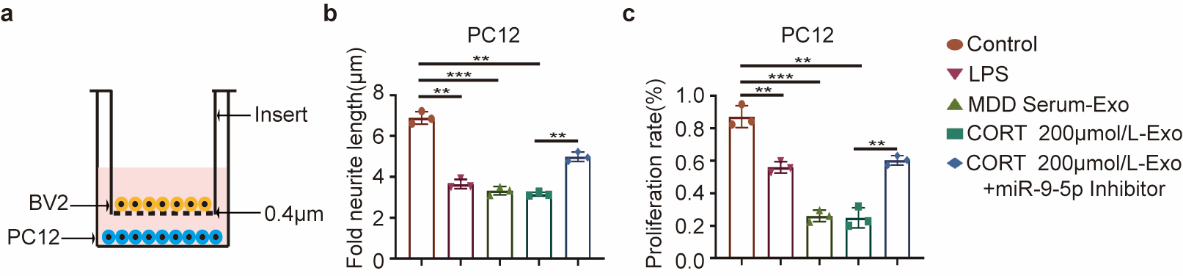


**Fig.S6** **a** Schematic diagram of co-culture. BV2 microglia cells were implanted into the upper compartment and treated with different treatments. **b**,**c** Quantitative staining of β3-tubulin and EdU in PC12 cells co-cultured for 48h with BV2 cells treated as above.
